# Lessons of practices of community-based maternal and child health surveillance system during and post COVID-19 in Indonesia

**DOI:** 10.1080/16549716.2025.2547438

**Published:** 2025-08-28

**Authors:** Wahyu Septiono, Ferdinand Pangihutan Siagian, Lutfan Lazuardi, Samsriyaningsih Handayani, Sabarinah Prasetyo

**Affiliations:** a Department of Biostatistics and Population Studies, Faculty of Public Health, Universitas Indonesia, Depok, Indonesia; b Center for Health Research, Universitas Indonesia, Depok, Indonesia; c Department of Pharmacology and Therapy, Faculty of Medicine, Public Health and Nursing, Universitas Gadjah Mada, Yogyakarta, Indonesia; d Department of Public Health and Preventive Medicine, Faculty of Medicine, Universitas Airlangga, Surabaya, Indonesia

**Keywords:** Maternal health, child health, community, surveillance, COVID

## Abstract

**Background:**

Amidst the onset of the COVID-19 pandemic, crucial integrated service post for women and children at the community level in Indonesia experienced disruptions. This change resulted in shifts in maternal and child health (MCH) surveillance.

**Objectives:**

This research aims to examine lessons from Indonesia’s community-based maternal and child health surveillance practices amid the challenges posed by the COVID-19 pandemic.

**Methods:**

The study was conducted in Depok, West Java, an urban area in Indonesia. A total of 20 key informant interviews with communities and government officials were carried out between October and December 2022. Data was analysed through content analysis.

**Results:**

The COVID-19 emergence led to an increased community-based MCH surveillance due to a growing public awareness to protect communities. The informants, while all acknowledging the importance of MCH surveillance, showed varied perspectives, highlighting gaps in perception. Four strategies in community-based MCH surveillance, including digital technology utilization, door-to-door visits, oral communication for identifying MCH cases, and community gatherings, had a positive impact on reporting and response. However, the incorporation of technology posed challenges, such as the diverse skill levels among community health workers (CHWs) and the general habit of producing hard copies after data entry.

**Conclusion:**

While the practices had a positive impact, the findings highlight the need to mitigate the challenges in data reporting. Two approaches may include standardization of reporting practices by using a single MCH surveillance application that can be accessed by CHWs and training programs to bridge the proficiency gaps in digital technology.

## Paper context

- **Main results**: Community-based maternal and child health surveillance during COVID-19 in Indonesia, through digital tools, door-to-door visits, and word-of-mouth, proved effective in reaching underserved populations and improving timely reporting, largely due to the trusted role of community health workers.
- **Added knowledge**: This study highlights that while community health workers were central to maternal and child health surveillance, challenges such as technological limitations, unequal community participation, and reliance on outdated reporting methods impacted data accuracy and coverage.
- **Global health impact for policy action**: Strengthening community health workers capacity through digital training, integrating mobile reporting systems, and securing policy and research support are critical actions to enhance the resilience and scalability of community-based maternal and child health surveillance.

## Background

Since the start of the COVID-19 pandemic, essential healthcare services for women and children were disrupted due to prevention measures, leading to changes in disease rates in these groups. Reports indicate a significant rise in global maternal and child mortality rates, increasing by approximately 38.6% and 44.7% per month, respectively, across 118 low- and middle-income countries (LMICs) during the outbreak [1]. This surge is attributed to heightened healthcare demands and delayed medical interventions. Particularly, LMICs encountered greater impacts than high-income countries (HICs) due to inadequate capacity of healthcare systems [2]. To prevent unintended negative impacts, future emergency plans therefore must include innovative maternal and child health (MCH) monitoring and surveillance, aiming at monitoring the progress, including the reporting and documentation of maternal and child morbidity that may lead to mortality [3].

Access to essential services for MCH such as immunizations, antenatal care, and nutrition programs underwent interruptions during the COVID-19 outbreak, potentially contributing to a rise in preventable diseases and maternal complications. Between April and August 2020, comprehensive phone surveys carried out in 39 LMICs revealed a significant portion of households refrained from or overlooked essential healthcare [4]. Ahmed et al. (2022) found a significant drop in MCH service use across 18 LMICs during the COVID-19 pandemic, directly linking it to a 1.5% rise in maternal mortality and a 3.6% increase in child mortality [5]. The implementation of physical movement restrictions amidst the COVID-19 outbreak impeded access to family planning services in India, Nepal, and South Africa [6–8], potentially leading to an increase in unintended pregnancies and poor pregnancy care.

Indonesia, an LMIC, operates a complex healthcare system. Community health centres (*pusat kesehatan masyarakat* in Indonesia term, abbreviated Puskesmas) are primary care hubs at the subdistrict level, also offering an extensive range of preventive and basic curative MCH care [9,10]. Each community health centre supervises some integrated service posts (*pos pelayanan terpadu* in Indonesia term, abbreviated Posyandu), a community-based health practice which is integral to Indonesia’s community health endeavours, pivotal in delivering vital MCH services [10–12]. Posyandu ensures antenatal care for expectant mothers, including checkups, nutrition guidance, and pregnancy information [10–12]. It also provides postnatal care for mothers and infants, encompassing breastfeeding support, immunizations, and growth monitoring [11,12]. Both puskesmas and Posyandu are strategically designed to cater to Indonesia’s unique healthcare needs for pregnant women and children, fostering health and early development.

The challenges of managing both COVID-19 cases and maintaining MCH services in Indonesia were overwhelming during COVID-19 pandemic. A study conducted in East Java Province found that the mortality rate among expectant women infected with COVID-19 was seven times higher than non-COVID-19 groups [13]. This could result in fear, generating a reduction in visits to MCH care. Studies in Indonesia revealed that the predominant cause for Indonesian women to abstain from seeking maternal health care during pregnancy and for health workers to perform such care was fear of COVID-19 infection [14–17]. During the initial stages of the outbreak, the attendance at Posyandu dropped markedly, primarily because of heightened anxiety and fear concerning the transmission of COVID-19 [18,19]. This disruption disproportionately affected vulnerable communities, where access to healthcare resources was already limited.

The pause in Posyandu operations emphasises the need for innovative strategies to maintain MCH monitoring and surveillance system during crises. Adjustments to MCH surveillance systems are essential to ensure uninterrupted services. This study focuses on investigating strategies and practices within Indonesia’s community-based MCH surveillance during the COVID-19 pandemic for lesson learnt. Specifically, this study aims to explore the practices implemented in community-based MCH surveillance systems (CBMCHSS) during the outbreak, examine how community engagement and outreach efforts evolved in response to the crisis, and identify innovations in MCH data collection and reporting that emerged during the pandemic.

## Methods

### Study area and research design

This study was performed in Depok, West Java, covering three urban villages across two subdistricts: Curug in Cimanggis subdistrict, Tirtajaya in Sukmajaya subdistrict, and Mekarjaya also in Sukmajaya subdistrict. In-depth interviews with specific type of informants were carried out in these locations. Individuals overseeing the MCH surveillance system at the subdistrict and urban village level and engaged in community-based surveillance received interview invitations through purposeful sampling. Informants were recruited, from the community health centre with responsibilities for MCH care and surveillance, subdistrict officials, and various community members who involved in MCH surveillance or was the beneficiary (i.e. community member). The research adopted a structured methodology, commencing at the community level to higher-level officials. Responses from community level informants were cross validated with the health centre, urban village, and subdistrict authorities, leading to an emergent study design where insights from earlier interviews influenced subsequent ones.

### Informant description

Out of 24 potential informants, 20 were successfully interviewed, representing diverse range of categories and locations. At urban village level, informants were distributed as follows: five (5) from Curug, five (5) from Tirtajaya, and four (4) from Mekarjaya. At the sub-district level, six (6) informants were interviewed at the district level, with three (3) from Cimanggis and three (3) from Sukmajaya. A total of 12 informants were categories under community-level roles, while the remaining eight (8) informants were categorized as government-level officials.

Five (5) types of informants engaged in MCH surveillance were identified at the community level. The CHWs, also known as *kader* Posyandu in Indonesia, played a vital role in promoting maternal and child health (MCH) at the local level through their involvement in the Posyandu program. Typically, they were responsible for conducting health checks, monitoring growth and development, providing health education, and supporting various health-related activities within the community. Secondly, the acronym ‘POKJA IV’ is an abbreviation for ‘*Pokja Penanggulangan Masalah Kesehatan Keluarga, Ibu dan Anak*’ in Indonesian. This translates to ‘Health Working Group (HWG) for Family, Maternal, and Child Health Problem Prevention’ in English. The responsibilities of this HWG primarily encompassed strategizing, executing, and overseeing involves planning, implementing, and monitoring health programs and interventions aimed at improving the well-being of families, mothers, and children within a specified area. The group collaborated closely with health authorities, community leaders, and other stakeholders to effectively accomplish its goals in advocating for family health and MCH. Thirdly, ‘*Tokoh Masyarakat*’ is an Indonesian phrase that can be rendered as ‘Community Figure’ in English. A community figure is a person who holds a position of respect and influence within a community. Fourthly, the term ‘Ketua RT’ is an abbreviation for ‘*Ketua Rukun Tetangga*’ in the Indonesian language, which can be translated as ‘The Neighborhood Association chairperson’ or a local leader in English. The position of Neighborhood Association chairperson entailed leading and overseeing the activities of the neighborhood association or community unit at the local level. Fifthly, community members were the beneficiaries of the MCH surveillance.

Four (4) categories of government officials engaged in MCH surveillance were identified. The Community Health Center (Puskesmas) Management officer was an individual responsible for strategic planning, coordination, and collaboration with other healthcare professionals and stakeholders to enhance the overall effectiveness of healthcare delivery at the community level. The community health centre surveillance officer typically was involved in monitoring and surveillance activities, including tracking and analysing health data, identifying trends, and implementing surveillance systems to monitor the health status of the community. They collaborated with other health professionals, CHWs, and local authorities to ensure a comprehensive approach to health surveillance within the community. Subdistrict officials worked under the local government and were involved in implementing government policies, managing local affairs, and coordinating activities that impacted the district’s development and welfare. Urban village officials encompassed various community engagement and coordination responsibilities within a specific territory at a lower level than a sub-district. These officers often worked under the local government and were involved in implementing community programs, managing local affairs, and facilitating communication between the community and higher government levels.

### Data collection

In-depth interviews were conducted from October to December 2022. A minimum of two investigators and a note-taker conducted in-person in-depth interviews lasting about 1.5 hours. Before the interviews began, all participants were provided with detailed information about the study objectives, procedures, and their rights as participants, including the voluntary nature of their participation and the right to withdraw at any time without penalty. Written informed consent was obtained from each informant prior to the interview. They were aware of the data collection process, which included recording interviews and transcribing the records. The data is accessible exclusively to the investigators. Informants received modest compensation for their time invested in interviews.

### Data analysis

This study employed qualitative content analysis (QCA) introduced by Graneheim and Lundmann [20,21]. Themes were established, and sub-themes emerging inductively during the analysis process to categorize analogous data illustrating primary discoveries related to the adaptations and inventive endeavours within maternal and child health surveillance amid the COVID-19 pandemic, ensuring optimal alignment with the research inquiries.

## Results

### Themes and sub-themes

Table 1 presents the themes and sub-themes derived from the content analysis. The qualitative data resulted in the identification of several corresponding sub-themes in four main themes that emerged. The first theme, Involvement, includes sub-themes such as adaptation/motivation, perception, and attitude, reflecting the personal and contextual factors that influenced CBMCHSS. The second theme, Implementation and Innovation, captures the practical aspects of surveillance through sub-themes like practices, reporting mechanisms, and digital technology utilization. The third theme, Obstacles and Challenges, highlights both the barriers encountered and the perceived opportunities for improvement, including general obstacles and specific challenges that presented opportunities for growth. Lastly, the theme Impacts on Reporting Quality comprises sub-themes related to the outcomes of the surveillance process, particularly focusing on reporting effectiveness, accuracy, and timeliness.

**Table 1.** Themes and sub-themes carried out in the study.

Themes	Sub-themes
Involvement	Adaptation/motivation
	Perception
	Attitude
Implementation and innovation	Practices
	Reporting mechanisms
	Digital technology utilization
Obstacles and challenges	Obstacles
	Challenges (opportunity to grow)
Impacts on reporting quality	Reporting effectiveness
	Reporting accuracy
	Reporting timeliness

### Involvement

The CBMCHSS adaptation emerged during COVID-19 outbreak, initially developed as an extension of the broader COVID-19 surveillance efforts. The outbreak may have strengthened the collaboration in community-based surveillance on maternal and child health as Posyandu activities were interrupted. As the community’s concern for each other has increased during the COVID-19 pandemic, some community members voluntarily joined the local COVID-19 task force to address COVID-19-related issues and help other community members affected by COVID-19. This motivation has been utilized by the community health centre and local leaders to engage the community as part of the COVID-19 surveillance. Although the primary aim was to detect and report COVID-19, a small part of MCH surveillance was also integrated into COVID-19 surveillance, particularly for the pregnant women affected by COVID-19. Local leaders encouraged community members to report any symptoms or health issues to them and CHWs to obtain information about the health status of mothers and children.

At the beginning of the COVID pandemic, maternal and child health reports were delayed. Even from the health office, reporting only came in late or after a report from a community health worker about pregnant women with complications. Well… it’s understandable, no one really expected we would face a pandemic like this, and as a result, Posyandu activities were disrupted too. Informant 7.

The residents in this neighborhood care about one another because they are also looking out for their own families. Someone even took the initiative to create a WhatsApp group for reporting COVID cases, but it’s also often used to report if something happens to a mother or her child.*’*Informant 12.

The COVID-19 pandemic served as a turning point in recognizing the critical role of community-level health surveillance. Prior to the COVID-19 pandemic, the focus of the community health centre was primarily on preventive and educational health programs, and surveillance was not part of the institution’s main activities. The emergence of COVID-19 prompted the awareness that community health centres should be responsible for health issues reporting within the community. Therefore, MCH surveillance, in addition to other diseases such as dengue, hypertension, illness related to environmental health, was strengthened through community-based practices. The CHWs therefore received reporting training to have adequate skill in surveillance at community level. They became the frontliners on reporting, in which data from CHWs was highly used by health centres.

The COVID-19 pandemic made us realize the true purpose of Puskesmas. Starting from the city level and the city’s Health Office, it became clear that we genuinely need specialists in maternal and child health surveillance. Informant 7.

Before COVID, we only focused on the preventive program, and we did not have staff to do surveillance Informant 8.

Although all informants emphasized the importance of MCH surveillance, they exhibited varied perceptions towards it. CHWs and health centre officials acknowledged MCH surveillance as a form of reporting and monitoring the health status of children and pregnant women. HWG officials specifically characterized MCH surveillance as contributing to the monitoring of stunting. Community members, neighbourhood association leaders, urban village officials, and subdistrict officials perceived surveillance as government assistance provided during disease situations such as aiding high-risk pregnant women with helps and providing additional food to malnourished children. The community health centre officials believed surveillance was solely focused on collecting data on COVID-19 cases, but it has evolved to include reporting various diseases, including MCH issues. Before COVID-19, community-based MCH surveillance was non-existent and, typically, reports were gathered only from visits to the community health centre.

Before COVID, we only relied on the reporting flow from the community to the Puskesmas. We lacked understanding about surveillance. We only knew it for COVID. Informant 7.

The rise in community-based surveillance was driven by a growing societal awareness and a desire to protect communities from COVID-19. The community health centre and urban village officials emphasized the community’s significant social cohesion during the COVID-19 outbreak. Many community members collected aid from neighbours and helped specifically individuals who were diagnosed with COVID-19 and required isolation at their homes. Furthermore, the CHWs admitted that their involvement in CBMCHSS was motivated by an intention to interact with the community, positioning themselves not only as the frontliners of MCH programs but also as essential members of the communities they assist.

I like to socialize, and I like being around people. As long as I can help my community, I will help them. Informant 14.

### Implementation and innovation

The reporting mechanism in the CBMCHSS adhered to a vertical pattern, and all informants had a thorough understanding of its procedure. Community members reported their health condition either to the CHW or to the neighbourhood association. The neighbourhood association chairperson who received reports directly from their community still reported to the CHWs. Afterwards, the CHWs submitted their reports to the health facilities, compiling all the information from their assigned region. The health centres subsequently transmitted the data to district health officials, who, in response, conducted any required follow-up actions based on the obtained information.

All data on maternal and child health is obtained from CHWs, either through Posyandu activities or direct reports from the community. Each month, every Posyandu reports its activities to the urban village office. After being compiled at the urban village level, the data is sent to the sub-district and Puskesmas. Every three months, the sub-district forwards the data to the Puskesmas and the City Health Office. Informant 1.

Four CBMCHSS practices emerged, encompassing the use of digital technology, door-to-door methodology, and oral communication (viva voce). The integration of digital technology involved CHWs and neighbourhood association chairperson collecting MCH data from households during periods of home isolation and self-distancing amid the COVID-19 pandemic, when traditional Posyandu services were disrupted. Similarly, in the context of COVID-19 and beyond, CHWs engaged in door-to-door visits to gather information and monitor the health of pregnant women and children. The post-COVID-19 period CBMCHSS saw a reliance on third-party sources, such as neighbours or other mothers, for MCH information when Posyandu attendance was low.

CHWs typically conduct visits from one household to another if they are aware of the presence of pregnant women there. Informant 4.

While purchasing groceries from the vegetable vendor, we inquire with pregnant women and mothers about any health-related concerns. Informant 3.

The utilization of mobile devices was incorporated into CBMCHSS practices. Some CHWs and neighbourhood association chief person utilized mobile applications for MCH surveillance through tools such as Google Forms, Google Sheets, and WhatsApp groups for receiving reports from community members and monitoring purposes. However, there were still some CHWs who favoured manual data collection (writing and noting). Using Google Forms and Google Sheets, pregnant women reported the progress of their pregnancies, and parents could report the development of their children. WhatsApp groups were also employed for MCH surveillance, where mothers can provide reports through this platform. Each pregnant mother was invited to join the WhatsApp group if their pregnancy was identified. Amidst the COVID-19 pandemic, a video call through WhatsApp was often employed to conduct a pregnancy check. In one health centre, a local university collaborates to develop a local version of a mobile MCH prototype, particularly for child growth monitoring. Moreover, the community health centre employs E-PPBGM and E-Cohort for reporting to higher policymaker levels, which are the platform used at national level.

We could not gather amid COVID, so we used WhatsApp video call Informant 6.

During my pregnancy, I received an invitation to join a WhatsApp group comprised of expectant women. Informant 17.

### Obstacles and challenges

Four obstacles were identified, including challenges in recruiting CHWs, administrative issues, socioeconomic inequalities within family groups, and social stigma. Recruiting CHWs for MCH surveillance was not an easy task due to the voluntary nature of the job. The requirement of dedicating time for reporting, monitoring, training, and regular meetings with health centres and low incentives for CHWs caused community members to hesitate in joining due to the high commitment involved. Administrative issues frequently hindered MCH data collection, such as cases where residents with temporary stays (renting) did not possess a local ID card (*Kartu Tanda Penduduk* abbreviated KTP) but have an original domicile KTP from another region, making their registration and monitoring often unreported. Some cases only came to light when, for example, pregnant women experienced severe health issues or when they had already passed away. Additionally, CHWs encountered challenges in collecting MCH data in affluent neighbourhoods, as this type of residents often prefer healthcare facilities and were not receptive to CHWs, considering CHWs only suitable for monitoring the health of the socioeconomically disadvantaged population. Finally, many mothers with children felt embarrassed or reluctant to report if their child was identified as stunted, resulting in underreporting. Furthermore, unmarried pregnant women often escaped monitoring due to social stigma and mothers received a stigma as well from CHWs if their child’s growth did not meet expectation and standard.

Many people here are non-residents who lease the property. They often do not report to CHWs. Informant 2.

CHWs frequently encounter challenges while attempting to engage with individuals residing in affluent households who also usually do not attend Posyandu. They are used to visit private clinics or hospitals. Informant 6.

When my son experienced only a slight increase in weight, I was approached by CHWs who questioned tendentiously, ‘Why is your child so thin? Do you not provide your child with milk? Or “Did you provide him with nutritionally deficient random food?” This situation causes me significant stress and feelings of shame.’ Informant 17.

The integration of technology utilization in CBMCHSS posed a dual challenge, manifesting in two distinct aspects. First, a visible disparity in computer-based or mobile application proficiency among CHWs was evident in the adoption of mobile applications. While some CHWs and the neighbourhood association chief person successfully incorporated mobile applications into their data collection practices, others lacked familiarity with such applications. Consequently, those who did not utilize mobile applications continued to rely on traditional paper documentation (e.g. manual entries in a logbook) for monitoring purposes, and subsequently submitted these records to the health centre. Second, even among those who employed mobile applications for data input or received self-reports from households, a common practice involved generating a hard copy of the report, which was then submitted to the health centre. Secondly, the reporting process to the national level involved numerous platforms, resulting in a cumbersome burden for health centre officials responsible for reporting duties.

The results of Posyandu were manually recorded by the CHW in a logbook, and this report was given to the health center. Informant 3.

CHW records our health symptoms and problems, enters them into the computer, prints reports, and delivers them to the health center. Informant 17.

### Impacts on reporting quality

The CBMCHSS practices showed a positive impact, particularly improving the reporting and the responsiveness of managing issues in MCH. For instance, the practice of direct home visits contributed to the registration of more pregnant women who were not enrolled in the National Health Insurance scheme. This led to a decrease in both maternal and newborn mortality rates in the community. Because of the discernible reporting procedure and coordination channels, reporting became more efficient and systematically organized. Furthermore, the regular reporting by CHWs expedited the process of data collection. Therefore, publics can anticipate receiving outcomes in a timely manner, and officials or policymakers at a higher level gave feedback more quickly. The CBMCHSS also could address issues of social stigma or humiliation that may have been felt by women facing financial difficulties.

The data collection of MCH issues can be faster, starting from the identification of cases to response. Informant 16.

In the past, the data collection process was merely focused on the gathering information, lacking subsequent follow-up actions. Now, there has been a noticeable improvement in the implementation of follow-up measures. Informant 4.

While community-based MCH surveillance enhanced the effectiveness of reporting, challenges persisted in the utilization of diverse platforms for transmitting data to the central level. The technology utilization such as cloud and mobile applications enabled direct reporting by the community. However, this effectiveness was constrained among CHWs who still maintained manual records or engaged in duplicating entries after the initial recording. Some CHWs even manually input data into computers, printed them, and then submitted them to the community health centre where the officials had to re-enter the information, creating an inefficient step. Additionally, while community-reported data proved effective, the reporting process to the central level using various platforms could be inefficient and time-consuming.

The Ministry of Health and BKKBN (The National Population and Family Planning Board) oversee numerous reporting platforms, including E-PPBGM, SIMPUS, SIBIMA, and New SIGA. There were moments when I ran out of time to enter data into every platform. Informant 20.

The CBMCHSS practice showed several inaccuracy issues. In some instances, stunting was misdiagnosed and measured incorrectly by CHWs, and re-evaluations revealed that it had not occurred. Furthermore, manual reporting by CHWs resulted in interpretation errors, as evidenced by the striking resemblance between the handwritten digits 5 and 6, which could lead to inaccurate statistics. Due to mistakes made during the input process, particularly when the community health centre had to re-enter data into the government platform, there was frequently a discrepancy between the information provided by the community health centre and the data entered by CHWs. This had a major effect on how accurately data was reported. Additionally, there were disparities in the data, especially for expectant and new mothers who reported directly to the community health centre rather than Posyandu, which led to gaps in the records.

Often, the community health centre receives data directly from the mothers without going through Posyandu. This generates gaps in data as the mothers and their infants go directly to the health centre. Informant 15.

If input manually, it is less accurate due to visual limitations, determining which one is correct between the numbers 5 and 6 can be challenging. Informant 5.

## Discussion

### Key findings

The adaptation of CBMCHSS during the COVID-19 pandemic in Indonesia significantly enhanced grassroots engagement, data collection, and health response mechanisms. The pandemic acted as a catalyst, shifting the role of community health centres and CHWs from primarily preventive education to active health surveillance, supported by increased social solidarity and local leadership. CBMCHSS evolved from informal community reporting to more structured systems involving CHWs, local leaders, and digital platforms, particularly as Posyandu activities were disrupted. Innovations such as digital platforms, door-to-door visits, and informal verbal reporting emerged to maintain surveillance amid disrupted Posyandu activities. However, challenges persisted, including disparities in CHW digital literacy, socio-economic barriers to data collection, underreporting due to stigma, and administrative burdens due to fragmented national reporting systems. While CBMCHSS improved the timeliness and responsiveness of MCH reporting, leading to earlier identification of at-risk pregnant women and children, it also revealed issues of data inaccuracy, reporting inefficiencies, and gaps between community-level and formal health facility records. These findings highlight the need for better integration of technology, sustained CHW training, and harmonized data systems to strengthen MCH surveillance at the community level.

### Interpretation of the results

The utilization of digital technology, door-to-door strategies, and word-of-mouth information emerged as community-based MCH surveillance strategies amid the COVID-19 outbreak, highlighting the importance role of CHWs in Indonesia’s surveillance system. CHW is the key success of conducting surveillance during the COVID-19 pandemic as demonstrated in Bhaumik’s study [22]. The community-based surveillance approach demonstrated efficiency, particularly as reporting was not solely reliant on a limited number of personnel in community health centre, who may find it challenging to reach community members. Consistent with findings from several studies, the use of digital technology at the community level represents an innovative effort, particularly in the reporting and surveillance of COVID-19 [23–25], with Kurniadi et al. (2021) found the widespread use of WhatsApp during the pandemic for maternal health surveillance in Semarang, Indonesia, during COVID [26]. Additionally, door-to-door visits by Community Health Workers (CHWs) became an alternative when Posyandu activities were halted, as observed by Helmyati et al. (2022) [17]. However, in the context of COVID, Paudel et al. (2022) discovered challenges in healthcare providers conducting door-to-door visits for MCH surveillance in Pakistan [27]. This contrasting outcome emphasizes the greater acceptance of CHW than healthcare providers within the community their high level of trustworthiness [28]. Word of mouth was also utilized by CHWs as an approach particularly for gathering information, acknowledging the positive and negative impacts affecting the MCH surveillance [29,30]. This has been found by Jalu et al. (2019), in which word of mouth proves to be effective in disseminating information sharing on MCH care in Ethiopia, but becomes a barrier when disseminating negative stigma [31].

This study discovered that community participation in MCH surveillance expanded into two viewpoints. Firstly, the majority of MCH surveillance during COVID-19 was dependent on CHWs who were already in place prior to the pandemic. As evidenced by a study conducted in Sierra Leone, where CHWs felt confident enough to help the response to COVID-19 due to their pre-pandemic experience and knowledge [32], our study found that involving CHWs in surveillance was not as difficult. Secondly, although MCH was initially integrated in COVID-19 surveillance, the involvement of other community members in the surveillance was motivated by a desire to protect the community by reducing COVID-19 transmission and severity including in mothers and children. This aligns with a study in Thailand by Singweratham (2023), which indicated that village health volunteer increased amid COVID-19 due to disease severity knowledge and preventative health behaviour [33]. Although community-based MCH surveillance practices are not governed by specific regulations in Indonesia, the existence of a national guideline for community health workers (CHWs), typically introduced during formal training provided by local health authorities, serves as an essential foundation. This guideline equips CHWs with basic knowledge of MCH-related issues, thereby enhancing their ability to recognize and respond to such concerns promptly within the community [34]. However, this study found that community-based MCH surveillance was easier to reach poorer than wealthier communities. Individuals in wealthier communities may hold certain stereotypes or biases due to misconceptions about the quality or expertise, or range of services, for example, provided by community-based health-related activities [35].

We found that community-based MCH surveillance significantly enhanced the collection of comprehensive data, improved the pace of reporting, and ensured timely responsiveness from policymakers. This approach demonstrated efficacy in reaching individuals who were previously unattainable through facility-based MCH data collecting. The presence of stigma frequently hinders seeking healthcare [36], thus necessitating a more thorough approach to data collecting through community monitoring rather than depending simply on healthcare facilities. Rwanda has effectively implemented a national digital health platform, RapidSMS, which enables CHWs to report MCH indicators via mobile technology. This system has contributed to more timely referrals and a notable reduction in maternal mortality rates [37]. Similarly, Bangladesh’s BRAC program integrates performance-based incentives with ongoing training and mobile health tools to enhance CHW motivation, improve data quality, and strengthen the accuracy of health reporting [38]. In addition, the limited healthcare facility workers, who may be burdened with heavy workloads amid COVID-19 [36,39], struggle to maintain continuous and thorough data gathering. However, this study highlights that the accuracy of data could be affected by the ineffective utilization of technology platforms and the continued use of old recording methods by certain CHWs, resulting in significant inaccuracies during data collecting and reporting. The utilization of technology, such as mobile applications that are integrated with healthcare facility systems, standardized, and user-friendly, can improve the accuracy of reporting [40,41]. Furthermore, continuous capacity building and digital literacy enhancement for CHWs and incentive frameworks that acknowledge and appropriately compensate CHWs for their essential contributions to MCH surveillance are encouraged to enhance the sustainability, efficiency, and responsiveness of CBMCHSS in both emergency and routine health contexts.

## Conclusions

The adoption of community-based MCH surveillance strategies has proven instrumental during the COVID-19 pandemic in Indonesia although challenges remained perceived. The COVID-19 pandemic revealed the critical need for resilient and adaptable surveillance systems at the community level. Our findings demonstrate how CBMCHSS practices, which were initially developed as an extension of COVID-19 surveillance, played a key role in maintaining MCH monitoring during a public health crisis. These practices were not only reactive responses but also offer replicable models for future emergencies. The integration of digital tools, door-to-door engagement, and informal reporting channels shows that low-resource settings could quickly adapt when regular MCH strategies were disrupted. Furthermore, investing in comprehensive training programs for CHW is paramount, focusing on enhancing their proficiency in utilizing digital technologies for data collection and reporting. Additionally, the integration of mobile applications with healthcare facility systems can streamline reporting processes and enhance overall efficiency and accuracy. Research initiatives to explore innovative technologies and methodologies, policy advocacy for governmental support, and capacity building for CHW networks further strengthen the foundation of community-based MCH surveillance, ensuring its resilience and efficacy.
